# Breast Cancer High-Penetrance Genes BRCA1 and BRCA2 Mutations Using Next-Generation Sequencing Among Iraqi Kurdish Women

**DOI:** 10.7759/cureus.62160

**Published:** 2024-06-11

**Authors:** Ahmad N Hassan, Mustafa S Mustafa

**Affiliations:** 1 Department of Medical Laboratory Technology, Erbil Technical Health and Medical College, Erbil Polytechnic University, Erbil, IRQ; 2 Department of Biology, College of Science, Salahaddin University-Erbil, Erbil, IRQ

**Keywords:** kurdish population, variants, ngs, brca2, brca1, breast cancer

## Abstract

Background

*BRCA1 *and *BRCA2 *genes are the main high-penetrance genes that are responsible for most cases of inherited breast cancer. The present study aimed to detect the frequencies of inherited breast cancer caused by *BRCA1* and *BRCA2* genes among Kurdish breast cancer patients, including all the exome of these two genes, using next-generation sequencing (NGS).

Methodology

Seventy women who were diagnosed with breast cancer and registered at Nanakali Hospital in Erbil, Iraq, were included. Blood samples were collected for molecular testing (polymerase chain reaction (PCR)) targeting all exomes of *BRCA1* and *BRCA2* genes. All exome regions are sequenced by NGS using the Miseq system (Illumina Inc., San Diego, CA). Obtained data were visualized using Integrative Genomics Viewer (IGV 2.3 Software, Broad Institute, Cambridge, MA). Data were interpreted based on the National Center for Biotechnology Information (NCBI), Clinically Relevant Variation (ClinVar) archives, and other databases.

Results

Among 70 samples, more than forty-two variants have been detected, 20 on *BRCA1* and 22 on *BRCA2*. Regarding clinical significance, six (14.28%) variants were pathogenic, four of them on the *BRCA1* gene, which were: c.3607C>T, c.3544C>T, c.68_69del, and c.224_227delAAAG, and two pathogenic variants were on *BRCA2* gene: c.100G>T, and c.1813delA. Also, two (4.76%) variants were conflict interpretations of pathogenicity, one (2.38%) was a variant of uncertain significant VUS, and the rest 29 (69%) variants were benign. In addition, four new variants (three in *BRCA1 *and one in *BRCA2 *gene), never previously reported, were identified.

Conclusions

In conclusion, analyzing the *BRCA1/2* genes provide a better prediction for the risk of developing breast cancer in the future. Variant types and frequencies differ among different populations and ethnicities, the common mutations worldwide may not be prevalent in the Kurdish population. The current research findings will be useful for future screening studies of these two genes in the Kurdish population.

## Introduction

Breast cancer is a type of cancer that forms in the cells and tissues of the breasts. It is the most common type of cancer among women, and it affects one in every eight to 10 women during their lifetime [1,2]. Breast cancer is caused mainly by non-genetic factors, while hereditary factors contribute to 5%-10% of the cases. Genetic factors refer to the inheritance of an abnormal (mutated) form of a susceptible gene; most inherited cases of this cancer result from mutations in genes that are linked to the breast [3,4].

*BRCA1/2* genes have expanded the knowledge of familial breast cancer, and *BRCA *genes are responsible for cell growth, division, and repair of damaged DNA. Their function is to keep the normal growth of breast, ovarian, and other cells. Altered forms of these genes cannot function normally and subsequently may lead to breast, ovarian, prostate, and colon cancer. In inherited breast cancer, these two genes are the most common causes; they may account for up to 10% of all cases [4-6].

Mutations in the *BRCA1* gene cause early-onset hereditary breast cancers with an estimated risk of 57% to 81% and cause hereditary ovarian cancers with an estimated risk of 90% in high-incidence families of breast and ovarian cancers [7]. Mutations in the *BRCA2* gene increase the lifetime risk by 45%-85%, while hereditary ovarian cancers have a lower risk than breast cancer [3]. Mutations in these two genes increase the risk and susceptibility of developing cancer, which is estimated to be up to 70% to 90% by the age of 70 [8,9]. The frequency and types of variants on these two genes differ among different populations and ethnicities. Information about different variations of the *BRCA1/2 *genes has a key role in the clinical diagnosis and management of different cancers resulting from them [10].

Hereditary breast cancer can be detected through genetic testing; advances in molecular genetics testing allow the detection of the abnormal breast cancer gene. The clinical approaches for high-risk individuals and their families have changed rapidly due to the recent progress and implementation of next-generation sequencing (NGS) and multi-gene panel testing in the field of hereditary cancer [11,12]. With these new techniques, the identification and diagnosis of genes associated with inherited susceptibility to breast cancer became much easier. Genetic testing plays a crucial role because early diagnosis of inherited abnormal genes related to breast cancer is important in the management of the disease [12,13].

Understanding *BRCA1/2 *mutations in any population provides a better risk assessment, prediction, and management of breast cancer. This cancer is the most common type of cancer in the Kurdistan region; the number of cases has been duplicated three times in the last decade [14]. This study aimed to detect the frequencies of inherited breast cancer resulting from mutations in the *BRCA1/2 *genes (including all the exomes) among Kurdish breast cancer patients in the Erbil Governorate.

## Materials and methods

Sample collection

A total of 70 samples that were diagnosed with breast cancer and registered at Nanakali Hospital for Blood Diseases and Cancer, Erbil, Iraq, were included. The blood samples were preserved in ethylenediaminetetraacetic acid (EDTA) tubes until further analysis. All participants were given informed consent, and after achieving their agreements, they were included as samples by the Helsinki Declaration.

Genomic DNA extraction

Genomic DNAs were obtained from each participant and isolated from 200 µl blood samples using the HiPure Blood DNA Mini Kit (Magen, China) following the manufacturer's instructions. NanoDrop (Thermo Scientific, Multiskan Sky-1530, Singapore) was used for estimating the quality and quantity of the extracted DNA. The DNA samples with (A260-A320) and (A280-A320) ratios, concentrations higher than 40 ng/μl were obtained.

*BRCA1* and *BRCA2* protocol

Primers were used for the coding regions (exons and the boundary intronic regions) of these two genes. There were 22 primers for the amplification of the *BRCA1* gene and 27 primers for the *BRCA2* gene at INTERGEN Genetics and Rare Diseases Diagnosis Research & Application Center, Ankara, Turkey.

Polymerase chain reactions (PCRs) were carried out for all samples using the designed primers for the isolated DNA samples, and PCR products were checked by agarose gel electrophoresis (2%), as shown in the Appendix (see Figures 2, 3). PCRs belonging to each participant were mixed to obtain PCR pools that have all the amplicons of each participant in one tube. During the mixing, the efficiency of the amplification and amplicon length were considered; the volume of each PCR is inversely proportional to the efficiency of the reaction and directly proportional to the amplicon length that was estimated based on gel electrophoresis. The NucleoFast® 96 PCR kit (Macherey-Nagel GmbH & Co. KG, Düren, Germany) was used for the purification of the PCR pools for each participant. These purified pools were quantified and standardized to 0.2 ng/ul, as required for the step of sample preparation. NexteraXT sample preparation kit (Illumina Inc.) is used for preparing the sample for NGS that is performed by the Miseq system (Illumina Inc., San Diego, CA).

Miseq alignment and read

The BWA-mem 0.7.17 was used for the alignment of the raw reads to hg19 [15]. Steps of sorting, duplicate marking, and base recalibration were carried out subsequently by Genome Analysis Toolkit 4 (GATK4) [16]. Variant Call was made using two separate algorithms, GATK UnifiedGenotyper and GATK HaplotypeCaller, which were both used to complement each other [16]. Using the GATK SelectVariants option and based on strand bias, read depth, and call quality parameters, low-quality variants from both sets were eliminated [16].

Mutation visualization and analysis

The data were visualized and read using Integrative Genomics Viewer (IGV 2.3 software, Broad Institute), the whole exome was analyzed, and for each detected change, variants were interpreted using NCBI ClinVar (https://www.ncbi.nlm.nih.gov/clinvar/) [17], BRCAExchange (https://brcaexchange.org/) [18], which was integrated with an international expert panel, the Evidence-Based Network for the Interpretation of Germline Mutant Allele (ENIGMA) consortium. Mutations with pathogenic, conflict interpretation of pathogenicity, and uncertain significance were assessed for the prediction of possible damaging effects using the MutationTaster changelog 2021 (https://www.genecascade.org/MutationTaster2021/) [19]. For *BRCA1 *gene (NM_007294.4) and *BRCA2 *gene (NM_000059.3, NM_000059.4) were used as reference sequences from the NCBI database (http://www.ncbi.nlm.nih.gov) [20].

Statistical analysis

*BRCA1/2* variants were compared based on their clinical consequences using the Fisher exact test. Data analysis was carried out using GraphPad Prism version 9.0.0 (121) (GraphPad Software LLC, San Diego, CA). A probability value of <0.05 was considered to indicate significance.

Ethical considerations 

The study was conducted according to the guidelines of the Declaration of Helsinki and approved by the Medical Ethics Committee of Erbil Polytechnic University (Approval No. 23-0011).

## Results

Among the 70 samples, more than 42 variants have been detected. Variants of intronic regions were neglected except for one variant that was not benign; finally, 42 distinct variants were included. In *BRCA1*, 20 variants were detected: 10 missense, three synonymous, two frameshift, and two nonsense variants were observed, plus three new variants. In *BRCA2*, 22 variants were detected: nine missense variants, eight synonymous, two nonsense, one frameshift, and one intronic variant, plus one new variant.

**Figure 1:**
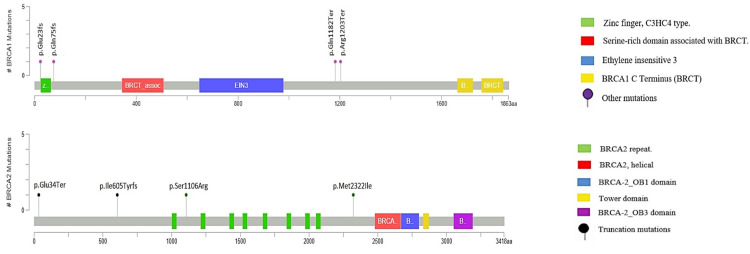
The schematic diagram of BRCA1 and BRCA2 protein changes with their positions according to the present study. Mutations on *BRCA1 *gene: p.Glu23fs, p.Glu75fs, p.Gln1182Ter, and p.Arg1203Ter. Mutations on *BRCA2 *gene: p.Glu34Ter, p.Ile605Tyrfs, p. Ser1106Arg, and p.Met2322Ile.

**Figure 2:**
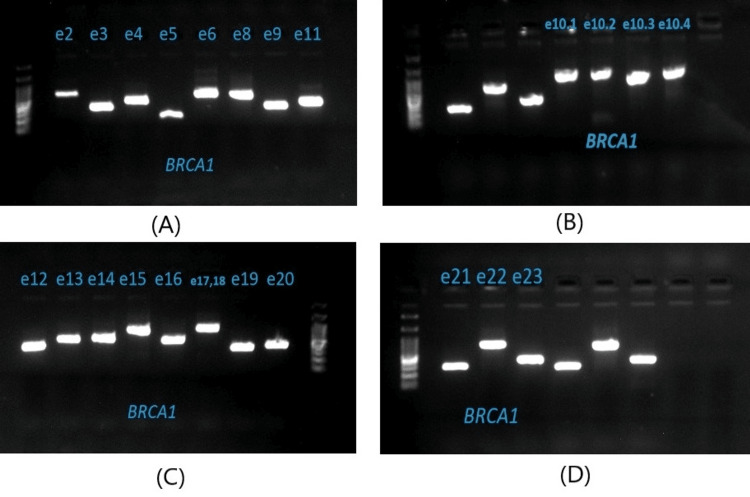
2% Gel electrophoresis for the PCR products of BRCA1 exons. (A): Exon 2 to 9, and 11. (B): Exon 10.1 to 10.4. (C): Exon 12 to 20. (D): Exon 21 to 23. PCR: polymerase chain reaction.

**Figure 3:**
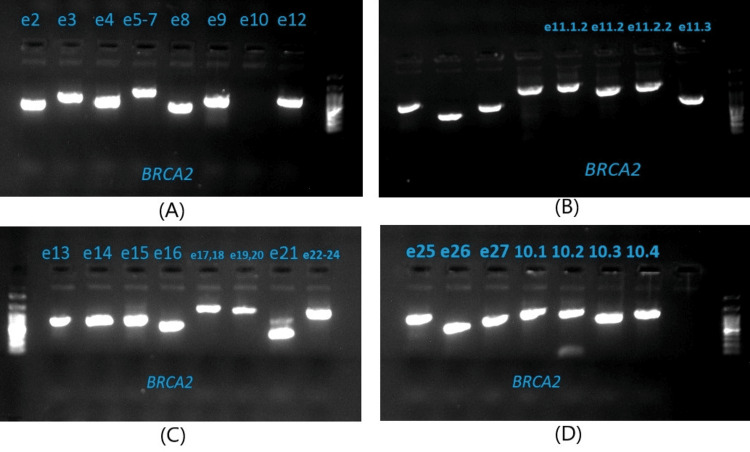
2% Gel electrophoresis for the PCR products of BRCA2 exons. (A) Exon 2 to 9, and exon 12. (B) Exon 11.1.2, 11.2, 11.2.2, and 11.3. (C) Exon 13 to 24. (D) Exon 25 to 27, exon 10.1,10.2, 10.3, and 10.4. PCR: polymerase chain reaction.

Among 42 variants, six of them were pathogenic, four of them on the *BRCA1 *gene, which were c.3607C>T, c.3544C>T, c.68_69del, and c.224_227delAAAG. The other two pathogenic variants (PVs) were on the *BRCA2* gene: c.100G>T and c.1813delA. All pathogenic variants were detected once among the 70 samples, with a case frequency of 1.43%. As shown in Table 1, regarding variants of conflict interpretations of pathogenicity, there were two variants, and both were on the *BRCA2* gene: c.1909+12delT and c.3318C>G. Also, one variant of uncertain significance was detected on the *BRCA2 *gene: c.6966G>T. Clinically important variants of *BRCA1/BRCA2* are shown in Figure 1.

**Table 1:** Variants of pathogenic, conflict interpretation of pathogenicity, and variant of uncertain significance (VUS) found on BRCA1/BRCA2 genes in breast cancer patients (n=70, the data has been represented as N, %). cDNA: copy DNA, AA: aminoacid, db SNP: single nucleotide polymorphism database, MAF: minor allele frequency, E: exon, Het: heterozygot, rs: reference SNP cluster ID, ENIGMA: Evidence-Based Network for the Interpretation of Germline Mutant Allele.

Variant				Case freq/zygosity	Mutation database		db SNP ID	MAF (min)	MAF (max)
Exon/Intron	cDNA	AA changes	Variant effect		ClinVar/NCBI	BRCAExchange/ENIGMA			
*BRCA1*: pathogenic variants									
E10	c.3607C>T	p.Arg1203Ter	Nonsense	1 (1.43%) Het	Pathogenic	Pathogenic	rs62625308	N/A	<0.01
E10	c.3544C>T	p.Gln1182Ter	Nonsense	1 (1.43%) Het	Pathogenic	Pathogenic	rs80357296	N/A	<0.01
E4	c.224_227delAAAG	p.Glu75fs	Frameshift	1 (1.43%) Het	Pathogenic	Pathogenic	rs80357697	N/A	N/A
E10	c.68_69del	p.Glu23fs	Frameshift	1 (1.43%) Het	Pathogenic	Pathogenic	rs80357914	N/A	0.01
*BRCA2*: pathogenic, conflict interpretation of pathogenicity, and VUS variants									
E3	c.100G>T	p.Glu34Ter	Nonsense	1 (1.43%) Het	Pathogenic	Pathogenic	rs80358391	N/A	<0.01
E1	c.1813delA	p.Ile605Tyrfs	Frameshift	1 (1.43%) Het	Pathogenic	Pathogenic	rs80359306	N/A	0.01
Intronic	c.1909+12delT	-	Frameshift	44 (62.8%) Het	Conflicting interpretations of pathogenicity​	Not yet reviewed	rs276174816	N/A	0.15
E11	c.3318C>G	p. Ser1106Arg	Missense	1 (1.43%) Het	Conflicting interpretations of pathogenicity​	Not yet reviewed	rs1298550035	N/A	<0.01
E13	c.6966G>T	p.Met2322Ile	Missense	1 (1.43%) Het	Uncertain significance	Not yet reviewed	rs80358924	N/A	<0.01

Also, twenty-nine benign variants were detected, of which thirteen were on the *BRCA1 *gene and sixteen were on the *BRCA2 *gene, as shown in the Appendix (see Tables 2, 3). Finally, four new variants were detected, three of them on the *BRCA1 *gene and one on the *BRCA2 *gene. Among 42 distinct variants, thirty-six of them were single nucleotide variants (SNVs), five of them were deletion (del), and one was duplication insertion (dup).

## Discussion

The current study applied NGS to the whole exome of *BRCA1/2* genes, as they contribute to most cases of hereditary breast cancer. The present study detected six pathogenic variants (8.57%) among 70 participants, four (5.71%) in the *BRCA1 *gene and two (2.85%) in the *BRCA2 *gene. Detecting more pathogenic variants in *BRCA1 *than *BRCA2* has also been proven by previous studies. The current study applied NGS to the whole exome of *BRCA1/2 *genes, as they contribute are contributing to most cases of hereditary breast cancer. The present study detected six pathogenic variants (8.57%) among 70 participants: four (5.71%) in the *BRCA1 *gene and two (2.85%) in the *BRCA2 *gene. Detecting more pathogenic variants in *BRCA1 *than *BRCA2 *has been proven by previous studies also, a study carried out in Turkey by Geredeli and his colleagues detected 11 germline mutations in *BRCA1 *and eight in *BRCA2*. In Italy, Concolino and his colleagues detected 24 deleterious variants on *BRCA1 *and 13 on *BRCA2*. In Pakistan, a study carried out by Tariq and his colleagues detected seven variants on *BRCA1*, four pathogenic and three VUS, while on *BRCA2 *only three VUS detected [21-23].

Detecting only six PVs that are related to high-penetrance genes among 70 participants is comparable with rates found in previous studies that included other populations, and according to the standards, the percentage of breast cancer that results from mutations in high-penetrance genes usually ranges from 5% to 10% [24]. It is true that our findings are within the usual range, but we should note that the present study detected four new novel mutations that, especially two of them, could be pathogenic because one of them is deleterious and the other is duplication, which according to the ENIGMA classification can be considered pathogenic. If these two variants are added to the reported pathogenicity, the percentage will rise to 11.43%, which goes above the usual range. Also, it is worth mentioning that the present study included *BRCA1/2* genes only; it is true that these two genes are responsible for most breast cancer cases due to germline mutations, but we should not forget that there are other genes that contribute to this type of breast cancer, and if they are investigated, this percentage may increase more.

According to the present study, the percentage of breast cancer cases due to germline mutations somehow goes along with the normal range worldwide, but unfortunately, cases of breast cancer in Erbil city increased dramatically, only between 2013 and 2019, the number of cases increased about three times, from 675 to 1884 in 2019 [14]. According to the same research, they revealed that the percentage of cases is predicted to increase during the present decade from 107.4% to 234.3% by 2030 in Erbil governorate. Based on these statistics, breast cancer is a main issue in this region. 

On the *BRCA2 *gene, two variants of conflict interpretation of pathogenicity (c.3318C>G), (c.1909+12delT), and one variant of uncertain significance (c.6966G>T) have been detected and reported previously on the ClinVar database. Such variants are somehow problematic and cause confusing for decision making by genetic counselors. Two of them, (c.3318C>G) and (c.6966G>T), are not even yet reviewed by some databases like BRCAExchange and ENIGMA. For understanding that, it is important to know that the classification of the variants regarding their clinical significance is changeable, and they depend on the submitted research to the databases and the tools used for the analyses. In the future, artificial intelligence (AI) may be used more efficiently for making more precise decisions [25,26].

The current study reported four new mutations that were never reported on the *BRCA *genes in any databases before, so they can be reported as novel variants. Three novel variants have been detected on the *BRCA1* gene: (c.3190A>C), (c.463dupC), and (c.981del), while on the *BRCA2 *gene, one novel variant has been detected: (c.3787A>G). Detection of new variants is normal because mutations of these two genes vary depending on geographical origin, population, and ethnicity, as has been proven previously [27,28]. Further analysis and investigations using bioinformatics tools and family history are required to estimate the clinical significance of these novel variants.

Finally, many *BRCA1/2* variants have been detected worldwide, and there is huge data regarding this issue around the world. Unfortunately, very little is known regarding these two genes among the Kurdish population. The present study is perhaps the first one carried out among Kurdish women using NGS looking for breast cancer cases due to germline mutations. It is true that this is a strong point for the current study, but we cannot compare it to previous data and research on our population. Until the time of performing this study, there were no NGS techniques in Erbil city, so we were obliged to transfer our practical work to Turkey, also, limited time, resources, and non-funding led to a small sample size.

## Conclusions

Molecular screening using NGS, and bioinformatics tools provides important information about hereditary types of breast cancer. Having information for those who inherited pathogenic variants is helpful in the prediagnosis of BC among relatives and those who are at risk for getting the disease. The percentage of pathogenic variants among Kurdish women is lower compared to other populations. The prevalence and type of variants differ among different populations and ethnic groups, also, new, and novel variants could be detected among various ethnicities. Pretests and routine screening are recommended for all women, especially those over forty years of age. Further studies are needed, including a larger sample size and other related genes to breast cancer, to better understand hereditary breast cancer among Kurdish women.

## Appendices

**Table 2:** Benign variants found on BRCA1/BRCA2 genes in breast cancer patients (n=70, the data has been represented as N, %). cDNA: copy DNA, AA: aminoacid, db SNP: single nucleotide polymorphism database, MAF: minor allele frequency, E: exon, rs: reference SNP cluster ID.

Variant				Case freq	Mutation database		db SNP ID	MAF (min)	MAF (max)
Exon/Intron	cDNA	AA	Variant effect		ClinVar	BRCA exchange/ENIGMA			
BRCA1: benign variants									
E6	c.536A>G	p.Tyr179Cys	Missense	3 (4.28%)	Benign	Benign/little clinical significance	rs56187033	N/A	0.03
E10	c.1067A>G	p.Gln356Arg	Missense	10 (14.28%)	Benign	Benign/little clinical significance	rs1799950	N/A	0.08
E10	c.2077G>A	p.Asp693Asn	Missense	3 (4.28%)	Benign	Benign/little clinical significance	rs4986850	N/A	0.11
E10	c.2612C>T	p.Pro871Leu	Missense	38 (54.28%)	Benign	Benign/little clinical significance	rs799917	N/A	0.50
E10	c.2311T>C	p.Leu771=	Synonymous	38 (54.28%)	Benign	Benign/little clinical significance	rs16940	N/A	0.50
E10	c.3113A>G	p.Glu1038Gly	Missense	38 (54.28%)	Benign	Benign/little clinical significance	rs16941	N/A	0.50
E10	c.3548A>G	p.Lys1183Arg	Missense	38 (54.28%)	Benign	Benign/little clinical significance	rs16942	N/A	0.50
E10	c.2082C>T	p.Ser694=	Synonymous	38 (54.28%)	Benign	Benign/little clinical significance	rs1799949	N/A	0.50
E10	c.1648A>C	p.Asn550His	Missense	2 (2.85%)	Benign	Benign/little clinical significance	rs56012641	N/A	0.03
E11	c.4308T>C	p.Ser1436=	Synonymous	38 (54.28%)	Benign	Benign/little clinical significance	rs1060915	N/A	0.50
E15	c.4837A>G	p.Ser1613Gly	Missense	38 (54.28%)	Benign	Benign/little clinical significance	rs1799966	N/A	0.50
E15	c.4883T>C	p.Met1628Thr	Missense	1 (1.43%)	Benign	Benign/little clinical significance	rs4986854	N/A	0.05
E15	c.4956G>A	p.Met1652Ile	Missense	3 (4.28%)	Benign	Benign/little clinical significance	rs1799967	N/A	0.06
BRCA2: Benign variants									
E10	c.865A>C	p.Asn289His	Missense	7 (10%)	Benign	Benign/little clinical significance	rs766173	N/A	0.17
E10	c.1365A>G	p.Ser455=	Synonymous	7 (10%)	Benign	Benign/little clinical significance	rs1801439	N/A	0.17
E10	c.1114A>C	p.Asn372His	Missense	35 (50%)	Benign	Benign/little clinical significance	rs144848	N/A	0.40
E11	c.2971A>G	p.Asn991Asp	Missense	7 (10%)	Benign	Benign/little clinical significance	rs1799944	N/A	0.17
E11	c.3807T>C	p.Val1269=	Synonymous	25 (35.7%)	Benign	Benign/little clinical significance	rs543304	N/A	0.28
E11	c.3055C>G	p.Leu1019Val	Missense	1 (1.43%)	Benign	Benign/little clinical significance	rs55638633	N/A	< 0.01
E11	c.5199C>T	p.Ser1733=	Synonymous	1 (1.43%)	Benign	Benign/little clinical significance	rs28897734	N/A	0.01
E11	c.4563A>G	p.Leu1521=	Synonymous	70 (100%)	Benign	Benign/little clinical significance	rs206075	N/A	0.13
E11	c.6513G>C	p.Val2171=	Synonymous	70 (100%)	Benign	Benign/little clinical significance	rs206076	N/A	0.13
E11	c.3396A>G	p.Lys1132=	Synonymous	25 (35.7%)	Benign	Benign/little clinical significance	rs1801406	N/A	0.48
E11	c.2229T>C	p.His743=	Synonymous	7 (10%)	Benign	Benign/little clinical significance	rs1801499	N/A	0.17
E14	c.7397T>C	p.Val2466Ala	Missense	70 (100%)	Benign	Not yet reviewed	rs169547	N/A	0.12
E14	c.7242A>G	p.Ser2414=	Synonymous	14 (20%)	Benign	Benign/little clinical significance	rs1799955	N/A	0.48
E18	c.8187G>T	p.Lys2729Asn	Missense	1 (1.43%)	Benign	Benign/little clinical significance	rs80359065	N/A	0.02
E22	c.8851G>A	p.Ala2951Thr	Missense	1 (1.43%)	Benign	Benign/little clinical significance	rs11571769	N/A	0.06
E27	c.9976A>T	p.Lys3326Ter	Nonsense	3 (4.28%)	Benign	Benign/little clinical significance	rs11571833	N/A	0.04

**Table 3:** Results of the NanoDrop spectrophotometer for the samples in the present study.

Sample no.	Sample code	DNA concentration ng/μL	260/280
1	D535	105	1.71
2	D536	201	1.77
3	D537	76	1.81
4	D538	106	1.72
5	D539	132	1.69
6	D440	83	1.72
7	D441	211	1.7
8	D442	96	1.68
9	D443	87	1.73
10	D444	106	1.82
11	D515	109	1.71
12	D516	261	1.69
13	D517	201	1.72
14	D518	208	1.7
15	D519	201	1.79
16	D520	153	1.81
17	D521	126	1.73
18	D522	174	1.78
19	D523	128	1.7
20	D524	98	1.69
23	D525	65	1.73
24	D526	109	1.78
25	D527	204	1.72
28	D528	106	1.8
30	D529	123	1.72
31	D530	261	1.73
32	D531	148	1.78
33	D532	129	1.7
34	D533	112	1.72
35	D534	109	1.72
36	Zh01	201	1.72
37	Zh02	109	1.78
38	Zh03	174	1.78
39	Zh04	211	1.7
40	Zh05	109	1.71
41	Zh06	105	1.78
42	Zh07	120	1.71
43	Zh08	150	1.8
44	Zh09	102	1.73
45	Zh10	202	1.81
46	Zh11	113	1.74
47	Zh12	89	1.76
49	Zh13	208	1.83
50	Zh14	254	1.73
51	Zh15	91	1.7
52	Zh16	212	1.82
53	Zh17	85	1.71
54	Zh18	206	1.78
56	Zh19	135	1.9
57	Zh20	160	1.72
58	Zh21	174	1.78
59	Zh22	151	1.9
60	Zh23	112	1.74
61	Zh24	149	1.86
62	Zh25	88	1.72
63	Zh26	156	1.8
64	Zh27	91	1.69
65	Zh28	135	1.76
66	Zh29	111	1.74
67	Zh30	95	1.8
68	Zh31	102	1.74
69	Zh32	79	1.72
70	Zh33	201	1.86
71	Zh34	105	1.72
73	Zh35	126	1.72
74	Zh36	113	1.8
75	Zh37	98	1.76
76	Zh38	220	1.9
77	Zh39	105	1.74
78	Zh40	176	1.82
